# Metabolic profiles and fingerprints for the investigation of the influence of nitisinone on the metabolism of the yeast *Saccharomyces cerevisiae*

**DOI:** 10.1038/s41598-023-28335-3

**Published:** 2023-01-26

**Authors:** Hanna Barchanska, Joanna Płonka, Paulina Nowak, Marianna Kostina-Bednarz

**Affiliations:** 1 Department of Inorganic Chemistry, Analytical Chemistry and Electrochemistry, Faculty of Chemistry, Silesian University of Technology, B. Krzywoustego 6, 44-100 Gliwice, Poland; 2 Biotechnology Centre, Silesian University of Technology, B. Krzywoustego 6, 44-100 Gliwice, Poland

**Keywords:** Diseases, Chemistry

## Abstract

Nitisinone (2-(2-nitro-4-trifluoromethylbenzoyl)-1,3-cyclohexanedione, NTBC) is considered a potentially effective drug for the treatment of various metabolic diseases associated with disorders of l-tyrosine metabolism however, side-effects impede its widespread use. This work aimed to broaden the knowledge of the influence of NTBC and its metabolites 2-amino-4-(trifluoromethyl)benzoic acid (ATFA), 2-nitro-4-(trifluoromethyl)benzoic acid (NTFA), and cyclohexane-1,3-dione (CHD) on the catabolism of l-tyrosine and other endogenous compounds in *Saccharomyces cerevisiae.* Based on a targeted analysis performed by LC–ESI–MS/MS, based on multiple reaction monitoring, it was found that the dissipation kinetics of the parent compound and its metabolites are compatible with a first-order reaction mechanism. Moreover, it has been proven that formed NTBC metabolites, such as CHD, cause a decrease in l-tyrosine, l-tryptophan, and l-phenylalanine concentrations by about 34%, 59% and 51%, respectively, compared to the untreated model organism. The overall changes in the metabolism of yeast exposed to NTBC or its derivatives were evaluated by non-targeted analysis via LC–ESI–MS/MS in the ion trap scanning mode. Based on principal components analysis, a statistically significant similarity between metabolic responses of yeast treated with ATFA or NTFA was observed. These findings facilitate further studies investigating the influence of NTBC on the human body and the mechanism of its action.

## Introduction

Humans generally consume more tyrosine (l-TYR) and phenylalanine (l-FE) as dietary protein than is required, and in excess, these amino acids are completely metabolized by the catabolic pathway. Tyrosine and phenylalanine are both glucogenic and ketogenic amino acids, mostly degraded in hepatocytes^1^. Amino acids and their metabolites play an important role not only as building blocks but also as precursors for neurotransmitters and hormones. In the catabolism of tyrosine, five enzymes are involved in the conversion of tyrosine to fumarate and acetoacetate, four of which are associated with recessively inherited metabolic disorders^2^.

A defect in tyrosine aminotransferase, the first enzyme of the tyrosine degradation pathway, leads to hereditary tyrosinemia type 2 (HT2), also known as the Richner-Hanhart syndrome. This is an inborn error of metabolism due to a block in the transamination reaction converting tyrosine to *p*-hydroxyphenylpyruvate, leading to elevated tyrosine levels in both blood and urine. The clinical phenotype of HT2 includes pseudodendritic keratitis, photophobia, and painful, palmoplantar, hyperkeratotic lesions^2,3^. The second step is performed by *p*-hydroxyphenylpyruvate dioxygenase (HPPD), which catalyzes the oxidation of 4-hydroxyphenylpyruvic acid to homogentisic acid (HGA). Mutations abolishing the HPPD function can lead to hereditary tyrosinemia type 3 (HT3), with characteristic features including intellectual disability, seizures, and periodic loss of balance and coordination or Hawkinsinuria—a rare condition associated with transient metabolic acidosis and hypertyrosinemia^4^.

Alkaptonuria is a rare genetic disease caused by the mutation of homogentisic acid oxidase, the third enzyme in the pathway that does not convert homogentisic acid to maleylacetoacetic acid. An increased homogentisic acid level in the circulation is a pathognomonic sign of ochronosis—the accumulation of ochronotic pigment polymer in various tissues that leads to systemic disease^5^. A defect in the last enzyme in the tyrosine degradation pathway, fumarylacetoacetate hydroxylase, causes tyrosinemia type 1 (HT1). This disorder leads to the accumulation of the toxic intermediates fumarylacetoacetate and maleylacetoacetate in body fluids and organs, which can induce acute organ failure and, in the long term, lead to carcinogenesis^2^. Other inborn errors of tyrosine metabolism include oculocutaneous albinism caused by a deficiency of melanin—synthesized by melanocytes from tyrosine in a melanosome, deficiency of tyrosine hydroxylase, the first enzyme in the synthesis of dopamine from tyrosine, and deficiency of aromatic l-amino acid decarboxylase, which also affects tryptophan (l-TRF) metabolism^6^.

The prognosis for people with hereditary tyrosinemia type 1 has changed dramatically with the introduction of a new treatment option called nitisinone (2-(2-nitro-4-trifluoromethylbenzoyl)-1,3-cyclohexanedione, NTBC), and for the first time in history, patients living with the disease are now reaching adulthood^7^. Since 1992, nitisinone—a compound derived from leptospermone produced by the bottlebrush plant (*Callistemon citrinus*)—has been an effective pharmacological treatment that rapidly and reversibly binds 4-hydroxyphenylpyruvate dioxygenase, which catalyzes the formation of homogentisic acid from 4-hydroxyphenylpyruvate^8^.

**Figure 1:**
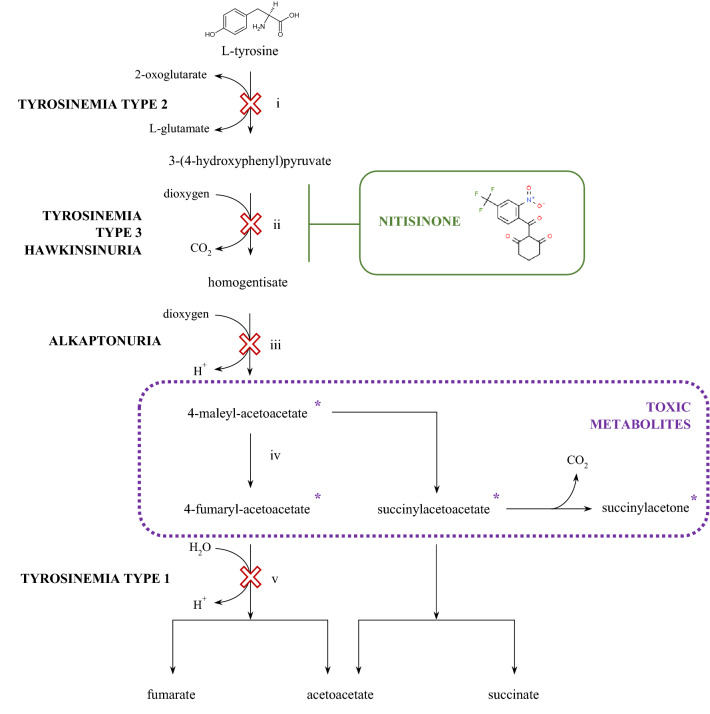
Tyrosine metabolic pathway highlighting the enzyme defects responsible and the site of action of nitisinone. i—tyrosine aminotransferase, ii—4-hydroxyphenylpyruvate dioxygenase, iii—homogentisate 1,2-dioxygenase, iv—maleylacetoacetate isomerase, v—fumarylacetoacetate hydrolase.

The use of NTBC in hereditary tyrosinemia is now almost established^7^, but there are other diseases where the potential role is still investigated, and high hopes are placed on it, such as neuroblastoma and oculocutaneous albinism^6,8^. The clinical development of nitisinone for alkaptonuria is also underway^1^. However, the inhibition of HPPD not only prevents the conversion of hydroxyphenylpyruvate (HPPA) to HGA but also leads to the accumulation of metabolites in the vicinity of this reaction. The nitisinone terminates the pathway at a stage corresponding to the phenotype of HT3, therefore l-Tyr and 4-hydroxyphenylpyruvate accumulate in the plasma. Massive tyrosinemia resulting from HPPD inhibition leads to deleterious effects such as cytopenia and corneal keratopathy due to high tyrosine concentrations. The clinical implications of nitisinone use are uncertain^9,10^. Figure 1 presents a diagram of the tyrosine metabolic pathway, highlighting the enzyme defects responsible and the site of action of nitisinone.

When introduced into the organism, NTBC can be subjected to three metabolic pathways: amino acid conjugation, forming two metabolites known as Schiff base derivatives (gly-NTBC and *b*-ala-NTBC), hydroxylation, and hydrolysis^11^. So far, three main compounds that are formed by the chemical degradation of nitisinone have been identified: 2-amino-4-(trifluoromethyl)benzoic acid (ATFA), 2-nitro-4-(trifluoromethyl)benzoic acid (NTFA), and cyclohexane1,3-dione (CHD); however, their pharmacological properties have not yet been determined. The structures of these compounds and selected physico-chemical parameters are presented in Table 1SM in Supplementary Materials^11–13^.

The main tyrosine metabolites proximally associated with HPPD inhibition are hydroxyphenylpyruvate, phenylalanine, tyrosine, and hydroxyphenyllactate (HPLA)^5^. A thorough understanding of the changes in the tyrosine pathway during NTBC therapy will be useful to optimize the management of NTBC-induced tyrosinemia^1^. Knowledge of tyrosine-induced molecular changes that may explain the symptoms of nervous system disorders is scarce, and it is widely believed that l-tyrosine can interfere with the cholinergic system via the dopaminergic system, which can occur because it is responsible for catecholamine synthesis^14^.

During neuronal development, a well-organized set of neuromodulators and neurotransmitters is important to produce the necessary stimuli for the formation of synaptic contacts and structural refinement; therefore, the correct interaction of the cholinergic system with the dopaminergic system as a modulatory system is important^3^. A thorough investigation of the mechanism of action of nitisinone is therefore crucial due to existing concerns about changes in neurotransmitter metabolism as a result of hypertyrosinemia. The hypothesis presented was that NTBC-induced hypertyrosinemia is related to higher concentrations of 3-methoxytyramine, suggesting a change in peripheral catecholamine metabolism^15^.

Tryptophan and tyrosine metabolites are neurotransmitters that modulate synaptic plasticity, cognitive function, and neuronal activity. Tyrosine hydroxylase (TH) is an enzyme involved in the metabolism of amino acids and neurotransmitters. The l-tyrosine is converted by l-dihydroxyphenylalanine (l-DOPA) decarboxylase into *p*-tyramine, which is also a neuromodulator, or by tyrosine aminotransferase (TAT) and fumarylacetoacetate hydrolase (FAH) into fumarate^10^. Catecholamine neurotransmitters may also be synthesized by the hydroxylation of tyrosine to l-DOPA, which is then converted to dopamine as a result of catalysis by the enzyme DOPA decarboxylase^16^. Finally, dopamine can be converted into several catecholamines, which function as neurotransmitters in the central and peripheral nervous systems^17^. Tryptophan serves as a precursor for the synthesis of the neurotransmitters serotonin and tryptamine. All of these listed monoamines have a common activation mechanism mediated through G protein-coupled receptors (GPCRs)^10^.

According to the literature, there is an effect on serotonin metabolism in HT1 patients treated with nitisinone^18,19^. A decrease in 5-hydroxyindoleacetic acid (5-HIAA) concentrations was reported in cerebrospinal fluid, which was due to increased concentrations of tyrosine, which competed with tryptophan by decreasing the availability of tryptophan for intracerebral serotonin synthesis. It has also been observed that with an increase in tyrosine, there may be an inhibition of tryptophan hydroxylase activity, the rate-limiting step in the serotonin metabolism^20^.

**Figure 2:**
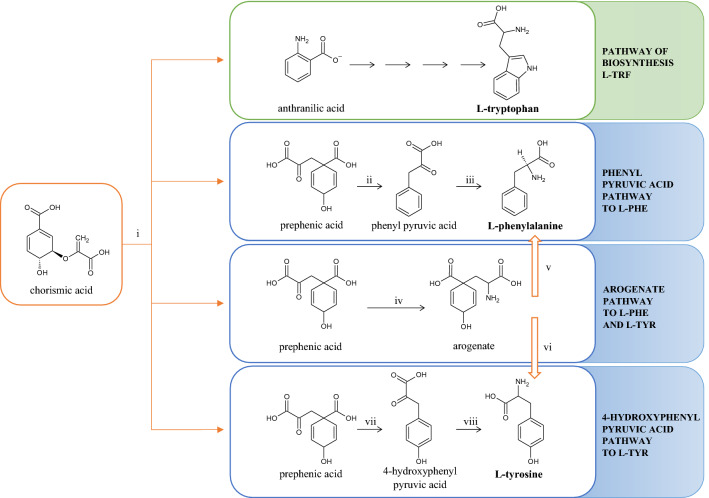
Biosynthesis of the aromatic amino acids tryptophan, tyrosine, and phenylalanine via a common pathway to chorismite in *S. cerevisiae*. i—chorismate mutase, ii—prephenate dehydratase, iii—transaminases, iv—transaminases, v—arogenate dehydratase, vi—arogenate dehydrogenase, vii—prephenate dehydrogenase, viii—transaminase.

In the human body, tyrosine can be formed from phenylalanine derived from the diet by the enzyme phenylalanine hydroxylase, found in large quantities in the liver. In the yeast *Saccharomyces cerevisiae*, tyrosine is produced through prephenate, an intermediate formed as part of the shikimate pathway^21^. The biosynthesis of aromatic amino acids in *Saccharomyces cerevisiae* follows the common aromatic pathway to chorismite—the unstable point that can partition into one of two pathways or branches. One path yields tryptophan while the other, via a Claisen rearrangement, yields prephenate, with is ultimately transformed into phenylalanine and tyrosine^22^. The tryptophan biosynthesis involves five steps: it starts with the conversion of chorismate to anthranilate, which is converted to TRF in four sequential steps by enzymes^23^. Tyrosine can be formed from arogenate or 4-hydroxyphenyl pyruvate, whereas phenylalanine may originate from prephenate passing through either arogenate or phenylpyruvate as intermediates^22^. A schematic pathway for the biosynthesis of tryptophan, phenylalanine, and tyrosine occurring in yeast is shown in Fig. 2^22–24^.

In light of the above, the purpose of this study was (i) to establish the kinetics of the dissipation of NTBC and its metabolites, (ii) based on metabolic profiles, to impact the influence of the drug and its derivatives on the catabolism of l-tyrosine, l-tryptophan, and l-phenylalanine, and (iii) based on fingerprints, to establish differences and similarities in the overall metabolic response of the model organism treated with NTBC and its derivatives. The results of the study provided new insights into the influence not only of NTBC but also of its metabolites on yeast metabolism, constituting a step forward in understanding the mechanism of action of nitisinone and, consequently, developing an effective therapy based on this drug.

## Materials and methods

### Chemicals and reagents

The 2-[2-nitro-4-(trifluoromethyl)benzoyl]cyclohexane-1,3-dione (NTBC), 2-amino-4 (trifluoromethyl) benzoic acid (ATFA), 2-nitro-4-(trifluoromethyl)benzoic acid (NTFA), and cyclohexane-1,3-dione (CHD) standards were supplied by Sigma Aldrich, Germany. Standard stock solutions with concentrations of 1.0 mg/mL were prepared in methanol. Analytical standards of l-phenylalanine (l-PHE), 5-hydroxy-l-tryptophan (5-HTRF), tryptamine (TRYP), l-tryptophan (l-TRF), dopamine (DA), dl-normetanephrine (NMN), dl-norepinephrine (NE), epinephrine (E), 5-hydroxytryptamine (5-HT), 5-hydroxyindole-3-acetic acid (5-HIAA), tyramine (TRA), l-tyrosine (l-TYR), 3,4-dihydroxy-l-phenylalanine (l-DOPA), *p*-coumaric acid (pCA), *trans*-cinnamic acid (tCA) and resveratrol (RES) (purity > 99%) were also purchased from Sigma Aldrich, Germany. Individual stock solutions with a concentration of 10.0 mg/mL were prepared in an aqueous solution of 0.05 M HCl with the addition of 5 g/L Na_2_S_2_O_5_, whereas the stock solution of pCA and tCA at the same concentration was prepared by dilution in methanol. All standard solutions were stored in amber glass vials at 4 °C in the dark. Working standard solutions were prepared daily by diluting the stock solutions in acetonitrile.

Acetonitrile (ACN), methanol (MeOH), formic acid (FA), acetic acid (AA), and water (LC–MS grade) were obtained from VWR, Germany. Analytical-grade Na_2_S_2_O_5_ and HCl (conc.) were obtained from STANLAB, Poland. Nylon syringe filters (0.45 µm, 25 mm, PURELAND, CHEMLAND, Poland) were used to filter the sample extracts. Pharmaceutical-grade glucose powder was supplied by Hasco-Lek S.A., Poland, and the yeast *Saccharomyces cerevisiae* strains were obtained from Lallemand, Poland.

### The model organism

As a model material for the experiments, the yeast *Saccharomyces cerevisiae* was used—a well-established model system for understanding fundamental cellular processes relevant to higher eukaryotic organisms^25^. *Saccharomyces cerevisiae* has been used as a model in many studies of the regulation of gene expression, cell cycle^26^, metabolism^27,28^, neurodegenerative disorders^29^, and other biological processes^25^. Despite their simplicity, yeast cells mostly possess the same basic cellular machinery as neurons in the brain, including pathways required for protein homeostasis and energy metabolism. Yeast is the first eukaryotic organism to have a sequenced genome that includes 6000 genes, with more than 60% of the genes already assigned a function. Significantly, approximately 40% of them have been shown, via comparative genomic analyses, to share conserved amino acid sequences with at least one known human protein^30^. Yeast has certain properties that make it particularly suitable for biological studies, including rapid growth and a well-defined genetic system; additionally, as a non-pathogenic organism, yeast can be handled with few precautions^31^.

### Sample preparation

#### Incubation conditions

Water (LC–MS grade) was added to the yeast at a proportion of 1 mL H_2_O per 5 g yeast and then homogenized. The yeast was exposed to NTBC and its metabolites separately at a concentration corresponding to the NTBC therapeutic dose (4 µg per gram of yeast). The treated material was incubated at 36 °C—a temperature similar to the human body. Glucose at concentrations of 1.2, 12.0, and 120.0 µg per gram of yeast was added to the samples to improve the metabolism of the model organism. Yeast not exposed to NTBC or its derivatives was carried out under the same conditions and constituted the blank sample. Each incubation experiment was performed in three independent replicates.

#### Sample preparation for the determination of NTBC and its metabolites and creation of yeast metabolic profiles

To demonstrate the effect of NTBC on selected yeast metabolic profiles, the quantitative analysis of NTBC, its metabolites, and selected endogenous compounds involved in l-TYR, l-TRF, and l-PHE metabolism was performed. Yeast was incubated for 5.5 h, with samples taken every half hour in 5.00 ± 0.01 g portions. The weighted material was treated with 5 mL of 8 mM FA in ACN and then shaken for 1 h at 500 rpm. Subsequently, each sample was filtered through a nylon filter with a pore diameter of 0.22 mm, and 1 mL of the clear extract was transferred to a vial for chromatographic analysis. Detailed information on the optimization of extraction conditions is available in Supplementary Material Section 1SM and Fig. 1SM. The blank and incremental samples for each analyte were analyzed in triplicate.

#### Sample preparation for the fingerprinting strategy

The yeast was incubated for 2 h, and samples were taken after 30 and 120 min. The homogenized yeast sample (1.0 ± 0.01 g) was mixed with 5.0 mL of methanol and then centrifuged for 20 min, 4000 rpm, at 20 °C. After centrifugation, the samples were filtered through a nylon filter (0.22 mm), and the obtained extract was diluted at 1:100, 1:1000, and 1:10,000 (v/v) in 8 mM FA in water immediately before analysis.

### LC–MS/MS instrumentation and conditions

The chromatograph used was a Dionex UPLC series (Dionex Corporation, Sunnyvale, CA, USA), coupled with an UltiMate 3000 RS (Rapid Separation) pump, an UltiMate 3000 autosampler, and an UltiMate 3000 column compartment with a thermostable column area. The entire configuration was controlled by the Dionex Chromeleon™ 6.8 software. Chromatographic separation of NTBC and its metabolites was carried out using the LiChroCART Purospher RP-18e column (125 × 3 mm, 5 mm, Merck), whereas metabolic profiling and fingerprinting were carried out on an TSKgel ODS-100 V (octadecyl, 150 mm × 4.6 mm, 5 mm particle size, TOSOH Bioscience, Tokyo, Japan). The flow rate and elution gradient were also optimized to minimize peak broadening. A binary gradient consisting of (A) 8 mM FA in ACN and (B) 8 mM FA in H_2_O was employed to achieve chromatographic separation. The final analytical conditions for the LC elution gradient and flow rate are described in Table 2SM in Supplementary Materials.

Mass spectrometric analyses were performed using an AB SCIEX 4000 Q TRAP triple quadrupole mass spectrometer (Applied Biosystems/MDS SCIEX, Foster City, CA, USA) equipped with an electrospray ionization (ESI) source. The Analyst 1.5.1 software (Applied Biosystems, Foster City, CA, USA) was applied for instrumental control, data acquisition, and quantitative analysis. The mass spectrometer was equipped with a Turbo Ion Spray™ ion source and was used in the positive and negative ion modes. The source-dependent operating parameters were optimized to obtain the best performance from the mass spectrometer for the analysis of compounds. The source-dependent parameters for targeted and non-targeted analyses were the nebulizer gas, the curtain gas, the collision gas, the ion spray voltage, and the temperature of the heater gas in the positive and negative modes, for details see Table 1.

**Table 1:** Source-dependent parameters for analysis.

Type of analysis	Ion spray voltage [V]	Source temperature [°C]	Nebuliser gas [psi]	Heater gas [psi]	Curtain gas [psi]
Determination of NTBC and its metabolites	− 4500	550	40	50	35
Determination of l-TYR, l-PHE, l-TRF and its metabolites	4500	450	30	35	35
	− 4000	450	30	30	35
Non-targeted analysis	4500	400	25	20	30
	− 4500	400	25	20	30

Based on reports^13,32,33^ on pharmaceutical and endogenous compounds in biological matrices, NTBC, as well as its metabolites and endogenous compounds, were determined by LC–ESI–MS/MS in MRM (Multiple Reaction Monitoring) mode, whilst non-targeted analysis was performed using EMS (Enhanced Mass Spectrometry) mode. Detection in Multiple Reaction Monitoring (MRM) using triple quadrupole and QTRAP LC–MS/MS systems offers superior selectivity because of double mass filtering in the mass analyzer. Therefore, MRM was used for targeted analyte screening and quantitation, where the analyte was detected and determined specifically through the combination of the parent mass and the unique fragments. The proposed method relies on unique parameters for each analyte to ascertain specificity which are molecular weight, generation of a specific fragment, and the HPLC retention time. The highest and second highest abundance transitions were used for quantification and confirmation, respectively. Because of the high selectivity of MRM detection, no matrix signals interfere with the quantification of the identified contaminant only affecting the intensity of the signals. Electrospray ionization was proposed for profiling of endogenous compounds since this ionization approach forms intact molecular ions. This selectivity greatly assists the screening for unknown and targeted analytes in complex samples matrix. The adjustment and optimization of the compound-dependent parameters declustering potential (DP), collision energy (CE), entrance potential (EP), and collision cell exit potential (CXP) were carried out by the direct infusion of a standard solution into the ion source, using a Harvard syringe pump at a flow rate of 10 µL/min. The compound parameters were optimized for each of the analytes and are represented in Table 2.

**Table 2:** Optimized MS/MS parameters for the determined compounds.

Analyte	Q1^a^ [m/z]	Q3^b^ [m/z]	DP^c^ [V]	EP^d^ [V]	CE^e^ [V]	CXP^f^ [V]
Positive ionization						
TRA	1381	103.2	46	9	29	6
		121.1	31		19	10
tCA	149.0	131.0	56	9	15	10
		103.1	56		29	6
DA	154.0	137.1	51	9	15	10
		91.2	31		19	10
TRYP	161.0	144.0	46	9	17	9
		115.0	46		43	8
pCA	165.0	146.9	56	9	15	10
		118.9	56		27	8
l-PHE	166.0	103.0	51	9	37	6
		120.5	51		19	8
NE	170.1	152.0	31	9	11	12
		107.1	31		19	10
5-HT	176.9	160.2	51	9	13	10
		115.1	31		39	8
l-TYR	182.0	136.0	51	9	19	10
		165.0	51		15	12
NMN	184.0	165.9	36	9	11	10
		134.0	36		25	10
E	184.0	166.0	41	9	15	12
		107.0	31		31	10
l-DOPA	197.8	152.0	51	9	19	12
		107.1	51		37	6
l-TRF	205.0	188.0	46	9	15	14
		146.0	46		25	10
5-HTRF	220.9	204.1	51	9	15	10
		162.0	51		27	12
Negative ionization						
NTBC	327.8	281.0	− 50	− 10	− 14	− 1
		238.6			− 40	− 1
ATFA	203.8	159.9	− 60	− 10	− 24	− 9
		119.9			− 40	− 7
NTFA	233.7	190.0	− 45	− 10	− 12	− 9
		160.0			− 20	− 9
CHD	110.8	69.0	− 55	− 10	− 24	− 7
		57.0			− 30	− 5
5-HIAA	189.9	145.9	− 60	− 10	− 16	− 9
		143.9			− 30	− 7
RES	226.8	184.9	− 90	− 10	− 28	− 9
		143.0			− 36	− 9

^a^Q1 Precursor ion, ^b^Q3 Fragment ion, ^c^DP Declustering potential, ^d^EP Entrance potential, ^e^CE Collision energy, ^f^CXP Cell exit potential.

Non-targeted analysis was conducted in the ion trap scanning mode enhanced mass spectrometry with positive and negative ionization. The Enhanced MS scan is the standard ion trap MS scan. In EMS, the ion trap is filled with molecular ions generated in the ion source, the ions are scanned out axially to the detector, to identify unknown compounds. Ions are transmitted from the source through the quadrupoles into the ion trap. This provides a full scan analysis of all the analytes entering that QTRAP system at that point in time. In the metabolic profiling and non-targeted strategy, chromatographic conditions were chosen to elute the entire spectrum of compounds characterized by different polarities. To achieve this, a TSKgel ODS-100 V column (octadecyl, 150 mm × 4.6 mm, particle size 5 μm) was used to separate complex samples with polar compounds with good peak shapes in a reversed-phase compatible with 100% aqueous eluents. This relatively long column allowed the analysis to run for 22 min, ensuring the detection of a large number of compounds from the test sample. The use of a gentle gradient mobile phase system starting with a solvent of low eluting strength and progressively changing to a solvent of higher strength aided chromatographic detection.

### Validation

**Figure 3:**
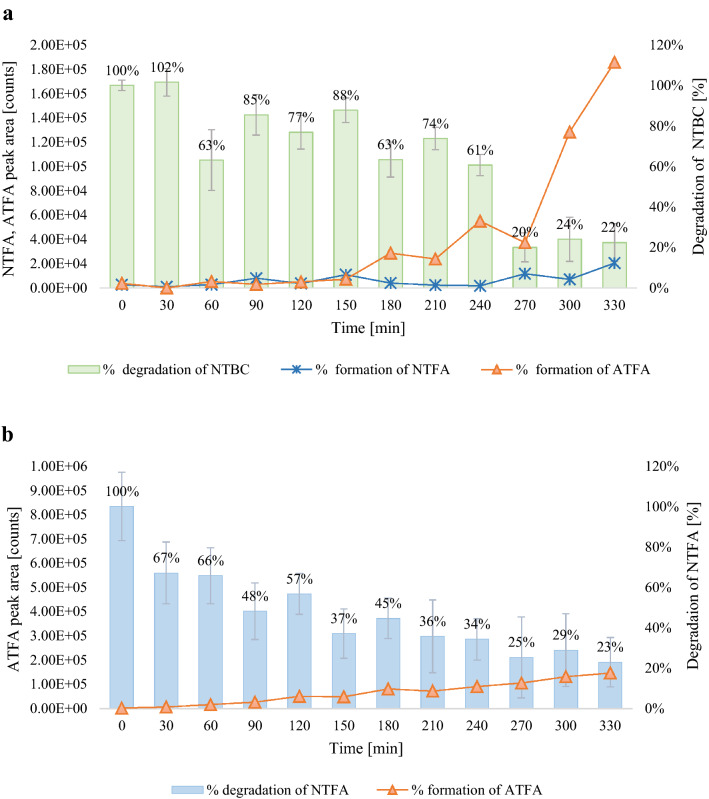
Degradation rate of (**a**) NTBC and (**b**) NTFA during incubation. Data are expressed as mean (n = 3); error bars represent CVs.

**Figure 4:**
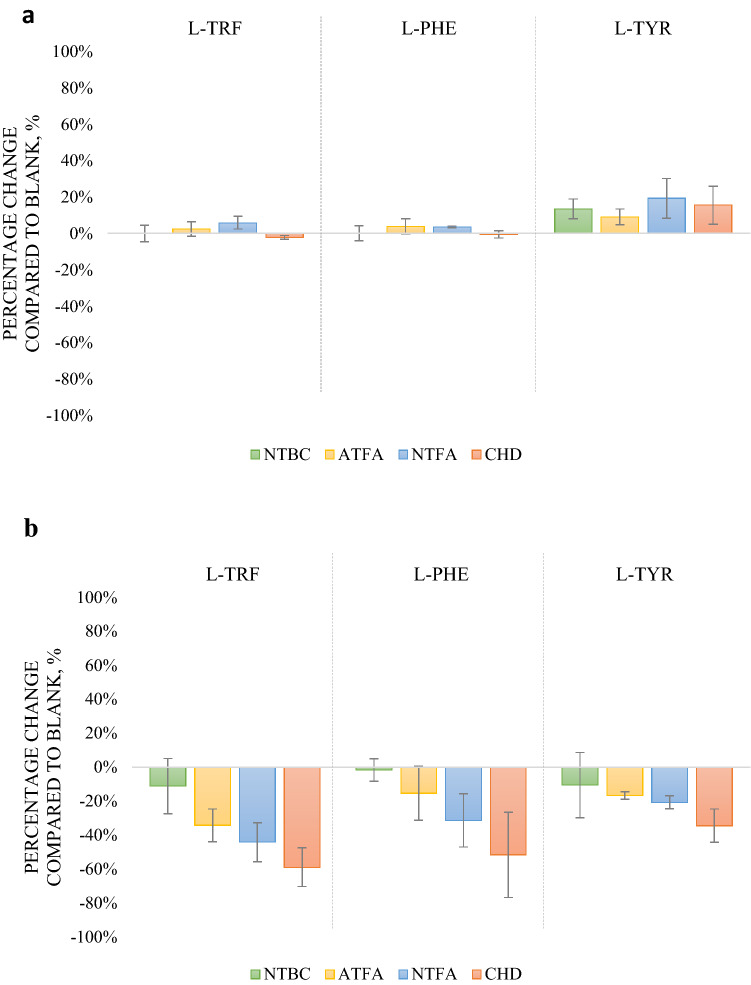
Percentage changes in the concentrations of metabolites of l-TYR, l-TRF, and l-PHE in yeast exposed to NTBC, NTFA, ATFA, and CHD after 30 min (**a**) and after 120 min of incubation (**b**). Blank was taken as 0%. Data are expressed as mean (n = 6); error bars represent CVs.

**Figure 5:**
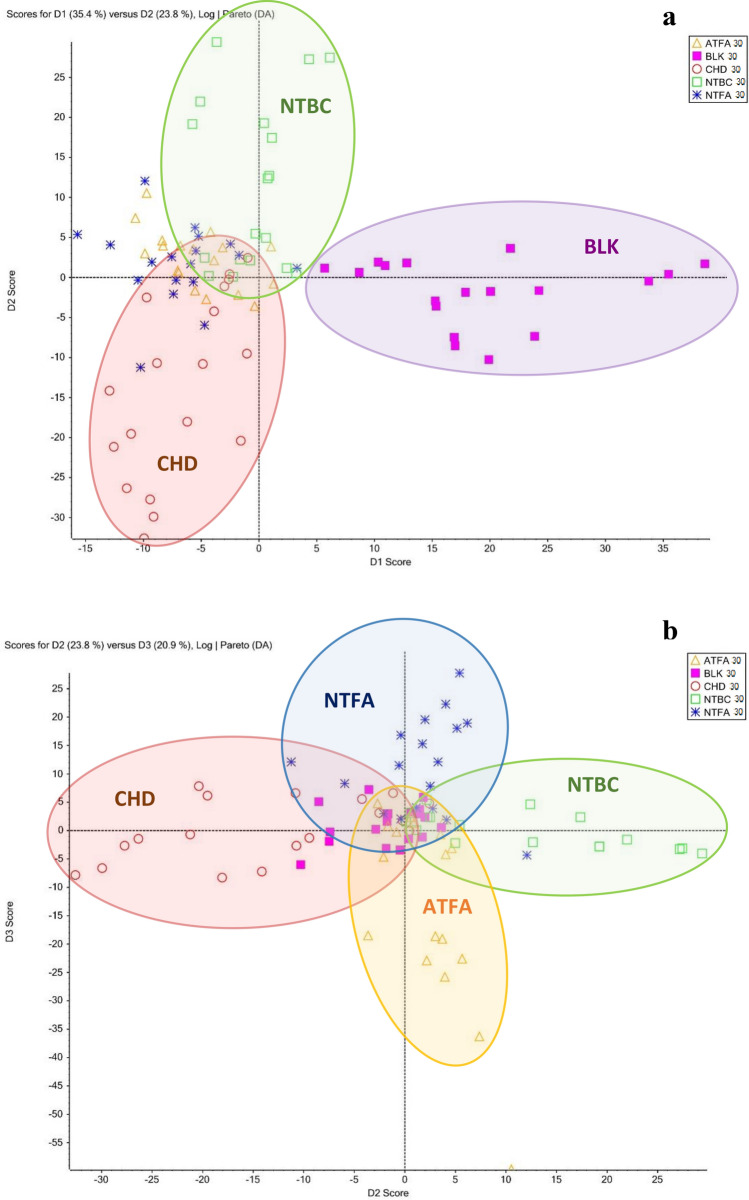
PCA plots for the data obtained after 30 min of yeast incubation of (**a**) the major components 1 (D1: 35.4%) and 2 (D2: 23.8%) and (**b**) the major components 2 (D2: 23.8%) and 3 (D3: 20.9%). Observations that can be associated with a variable group are delineated (ellipses).

The methods developed for the targeted analysis of NTBC and its metabolites in the model organism were validated for the compounds listed in Table 3SM in Supplementary Materials. The calibration curves were generated in acetonitrile, with three replications, in the concentration range of 0.2–8.0 mm /mL, whereas the matrix-matched calibration curves were prepared in the same way but in blank yeast extracts obtained under specific extraction conditions. The results were plotted on a graph of peak area-to-analyte concentration and are defined as linear over the selected working range because the linear regression models had a determination coefficient (R^2^) value of 0.99 for most analytes. The limits of determination (LOD) and limits of quantification (LOQ) were evaluated from the concentrations of each compound in signal-to-noise ratios of 3 and 10, respectively. The lowest concentration of a substance that was possible to be determined using this analytical procedure was in the range 0.0042–0.1779 μg/mL. Analyte recoveries were determined at three spiked compound concentration levels for the samples. The accuracy of the method was presented as a recovery value with a standard deviation. Precision indicated the reproducibility and it was determined as the percentage of the standard deviation from the arithmetic mean of the calculated sample concentrations and expressed as the percent of coefficient of variation (CV). The chromatograms of the blanks and spiked yeast samples were compared to identify any peaks during the analysis. The specificity test monitored multiple mass transitions and their relative intensities to evaluate any significant interference in the chromatograms. The matrix effect (ME) was calculated as a ratio of the slope of the calibration curve of the analyte in the mobile phase and in the blank sample extract (matrix-matched calibration plot). A matrix effect value of 100% indicates no effect, a value below 100% indicates an ionization suppression, and a value above 100% indicates an ionization enhancement due to the coeluting sample compounds. The final ME values, presented as percentages together with the other validation data, are shown in Table 3SM in Supplementary Materials. Chromatogram of the blank sample obtained by the analysis method for the determination of NTBC and its metabolites is presented in Fig. 2SM and chromatograms of a matrix solution spiked with a standard solution of NTBC and its metabolites are shown in Fig. 3SM in the Supplementary Material. The validation of the developed LC–MS/MS method was considered satisfactory. The selection of the assay conditions (extraction conditions, LC–MS parameters) of the developed methodologies is the result of a compromise between the high matrix complexity, its variations, the number of compounds to be determined in one analytical process in a short time, and the values of the validation parameters. Determination of selected endogenous compounds involved in the metabolism of l-TYR, l-TRF and l-PHE was performed by semi-quantitative analysis, by comparing changes in the content of these compounds in exposed samples compared to blank samples. Chromatogram of the blank sample obtained by the analysis method for the determination of l-TYR, l-TRF and l-FA is shown in Fig. 4SM, while the chromatograms of a matrix solution spiked with a standard solution of these analytes are presented in Figs. 5SM and 6SM in the Supplementary Material. The validation parameters are appropriate for the assumed objective as well as the determination of changes in the selected endogenous compounds in yeast exposed to NTBC and its derivatives.

### Post-acquisition data analysis

Statistical significance was determined using Student's *t*-test (*p* < 0.05). The software used to operate the mass spectrometer was Analyst (Version 1.5.1, Applied Biosystems, Foster City, CA). The programs LightSight (v. 2.2.1 Applied Biosystems, Foster City, CA) and MarkerView (v. 1.1, Sciex, US) were used for post-acquisition data processing.

## Results and discussion

### Degradation of NTBC and its metabolites

The term degradation kinetics refers to the study of the rate of drug degradation. Most pharmaceutical products are degraded through first-order reactions^34^. A series of experiments were performed according to the procedure described in “Incubation conditions”. for the determination of stability, the rate of disappearance, and to evaluate the metabolic efficiency of nitisinone and its metabolites. Degradation rate constants were calculated as values proportional to the decrease in the initial concentration of the drug as a function of time. The degradation kinetics were carried out using different exposed compounds (drug NTBC and its metabolites ATFA, NTFA, and CHD) and glucose solutions at various concentrations, and the data were plotted as a percentage of drug remaining vs. time. Graphs illustrating the progression of NTBC, NTFA, and ATFA metabolism after the addition of different amounts of glucose are shown in Fig. 7SM in Supplementary Material. As the CHD was stable under experimental conditions, the curves of this composure were not included. A glucose solution was added to the yeast samples at 1.2, 12.0, and 120.0 µg per gram of yeast to test the effect of glucose on the degradation process. It was supposed that increasing the concentration of glucose would accelerate the degradation process. However, the experiment showed that with high glucose concentrations, the metabolism was altered. If the glucose level is too low and the yeast has access to oxygen, alcoholic fermentation is carried out for a short time, and the sugar is completely used for aerobic respiration. After 1 h of incubation with the lowest glucose dose, the most intense yeast activity was observed, which is also shown by the decrease in compound concentration during this time. For each compound, this characteristic point was followed by a stabilization of the degradation curve, which is due to the weakening of the activity of the enzymes contained in the yeast and a decrease in the metabolic rate. In the experiment in which glucose was added at higher doses (12.0 and 120.0 µg per gram of yeast), the metabolic decrease occurred a little later, at 90 min, most likely because of a different form of respiration. A glucose amount of 12.0 µg per gram of yeast was taken for further investigations because the strongest metabolism occurred after the addition of this concentration. The average reaction rate constant was highest when glucose was added at a dose of 12.0 µg per gram of yeast. The values of the rate constants for each reaction are included in the graphs in Fig. 7SM in Supplementary Material. Under the conditions studied, the gradual formation of NTFA and ATFA was observed during the degradation of nitisinone; this correlation is shown in Fig. 3a. The levels of these metabolites initially increased at a similar rate, but after about 150 min, the concentration of ATFA was significantly higher than that of NTFA, probably due to the metabolism of NTFA to ATFA over time. This assumption is supported by an experiment in which the yeast was exposed only to NTFA, with the degradation of this compound with the formation of ATFA. The curve of the disappearance of NTFA with simultaneous ATFA formation is shown in Fig. 3b. Most importantly, NTFA was the most metabolized compound of all compounds tested. There might be various reasons for this rapid degradation. One possible explanation is that the compound is rapidly converted into an ATFA derivative, which is relatively stable. This study appears to be the first to find that the NTBC degradation produces its derivatives ATFA and NTFA, and yet additionally NTFA is metabolized to ATFA. Overall, these results emphasize the challenges in estimating the impact of NTBC on secondary metabolism due to the complex metabolic process of NTBC. These results are encouraging and may facilitate a significant step towards developing an effective nitisinone-based therapy, but they should be validated using a larger sample size.

### Metabolic profiling strategy

The changes in the concentrations of compounds involved in the metabolic pathways of l-TYR, l-PHE, and l-TRF were expressed as percentage changes in yeast samples exposed to NTBC, NTFA, ATFA, or CHD compared to the concentrations of these compounds in blank samples. Off all endogenous compounds included in this study, there were statistically significant changes (*t-*test at the significance level of 5%) in the concentrations of l-TYR, l-TRF, and l-PHE in yeast exposed to NTBC or its metabolites compared to the blank sample. Figure 4 shows the graphical visualization of the obtained results. No substantial changes were observed in the metabolic response of the l-tyrosine pathway of yeast exposed to NTBC for 30 min (Fig. 4a). This is in line with the assumption presented above, namely that NTBC metabolizes slowly during 30 min of incubation. Hence, it can be concluded that nitisinone does not induce metabolic effects in this model organism, even after 120 min of incubation. The situation is different when the changes induced by NTBC metabolites are considered. After 120 min of incubation, i.e., the time of the strongest yeast metabolite process, there was a significant decrease in the amino acid content, especially after CHD exposure (Fig. 4b). This indicates that CHD, as the most stable compound among these metabolites, has the strongest influence on the model organism, hence the greatest deviations in the concentrations of l-TYR, l-TRF, and l-PHE, whose contents, between 30 and 120 min of incubation, decreased by 50%, 51%, and 57%, respectively, in comparison to the blank sample. Initially, NTFA did not show a reducing effect on amino acids after 30 min of the experiment, but after a longer period, it had a negative effect on these compounds (concentration changes of l-TYR: − 40%, l-TRF: − 50%, l-PHE: − 35% between 30 and 120 min of incubation in respect to the blank sample). This may be due to the synergistic action of NTFA and ATFA, of which the latter is formed during the breakdown of NTFA. Hence, the effect of NTFA was stronger than that of ATFA, which, at the initial incubation stage (30 min), had a weak effect on the concentrations of amino acids, but after 120 min of incubation, the influence was higher by − 26% for l-TYR, − 37% for l-TRF, and − 19% for l-PHE. The metabolic effect induced by ATFA showed the same tendency as that induced by NTFA, only slightly weaker in potency.

### Metabolic fingerprinting strategy

The targeted analysis indicated the effects of nitisinone and its metabolites on the contents of three compounds involved in the tyrosine, tryptophan, and phenylalanine metabolism pathways, which normally occur in yeast. A non-targeted analysis was performed to reveal the global effects of NTBC, ATFA, NTFA, and CHD on the model organism. Following the procedure described in “Sample preparation for the fingerprinting strategy”, a series of matrix extracts was prepared. The model organism was exposed individually to NTBC, ATFA, NTFA, and CHD in three repetitions. Samples for chromatographic analysis were taken after 30 and 120 min of yeast incubation to determine the effects of the compounds over time on the metabolism of the model organism. Due to the complexity of the matrix and the difficult-to-predict concentrations of the compounds contained in the yeast, the analysis was performed at three dilution levels (1:100; 1:1,000; 1:10,000, v/v) in 8 mM FA in H_2_O to obtain the full spectrum of information available from these studies. Chemometric tools were used to process the resulting chromatographic mass data, using the MarkerView software.

Reducing the dimensionality of the dataset consisting of many variables correlated with each other while retaining the variation present in the dataset was obtained by using the dimension reduction algorithm. This was done via principal components analysis (PCA), a multivariate tool used to find the main source of variability present in the data sets. The main components were defined by the covariance matrix as the variables were standardized using the standard normal variate (SNV). To minimize the effects of small noisy variables on chemical data from the same analytical technique where the larger peaks are often more reliable and less susceptible to noise, Pareto scaling was used. The standardized data from chromatographic and mass analyses were subjected to linear transformation to convert the multivariate data into a form in which the variables are not correlated. Regression analysis provided a preliminary indication of whether there was a correlation between the responses of yeasts exposed to NTBC and their metabolites with respect to the blank sample during the incubation period. These new uncorrelated variables—principal components—were ordered in descending order according to the variance explained. Principal components analysis simplified the visualization of complex data sets for exploratory analysis, and the results were evaluated by examining score plots that showed the variable responses for all samples. Here, PCA was used as the main fingerprinting strategy that represented the relationship between the exposed compounds and the global metabolic response of the organism.

**Figure 6:**
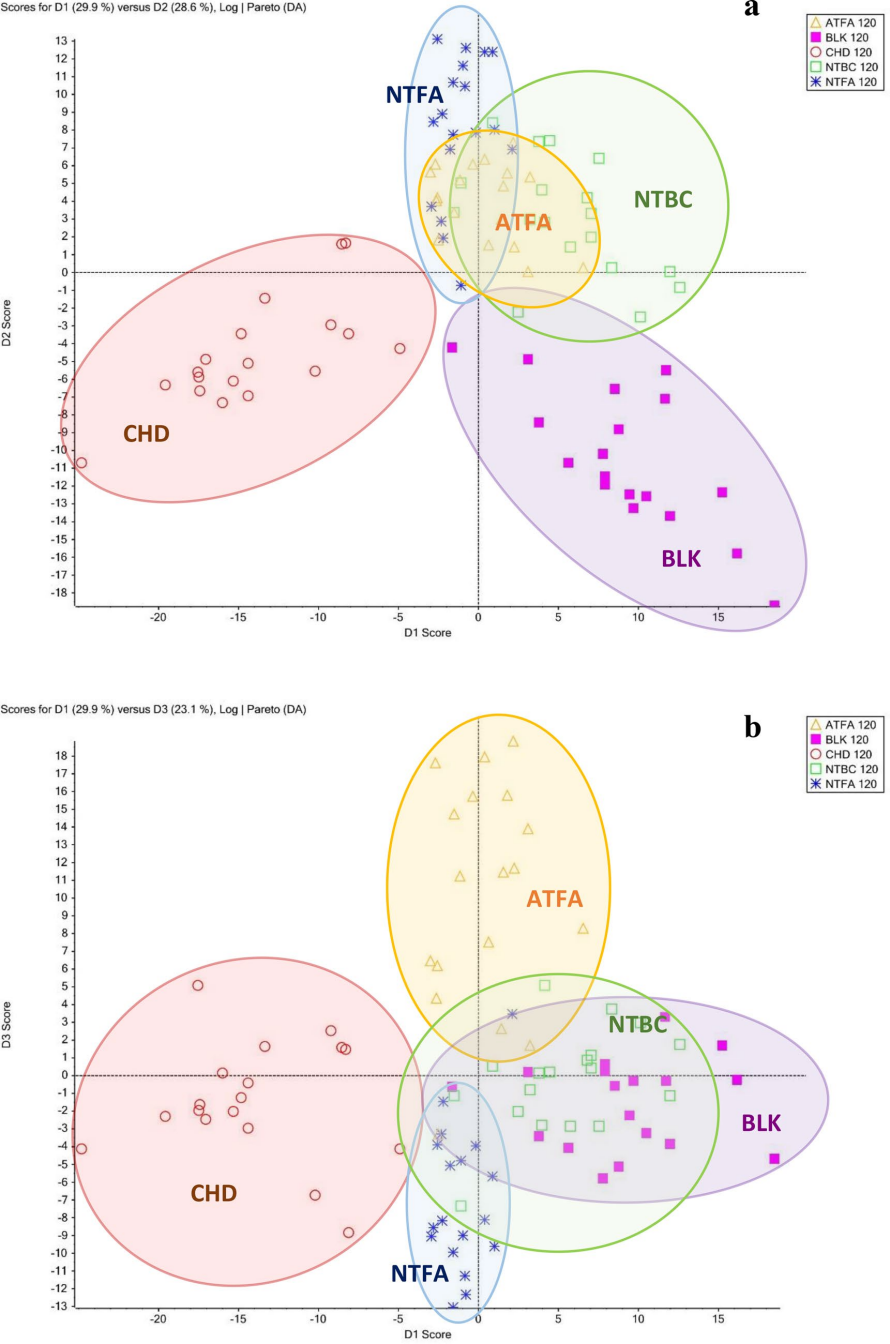
PCA plots for data obtained after 120 min of yeast incubation of (**a**) the major components 1 (D1: 29.9%) and 2 (D2: 28.6%) and (**b**) the major components 1 (D1: 29.9%) and 3 (D3: 23.1%). Observations that can be associated with a variable group are delineated (ellipses).

Statistical data processing proved that it was possible to identify the effect following the treatment of yeast with each compound (NTBC, ATFA, NTFA, and CHD) after 30 and 120 min of incubation in relation to the blank samples (Figs. 8SM and 9SM). This fingerprinting strategy provided an unambiguous identification of treatment with nitisinone or its metabolites in the model organism because of the marked metabolic response of the organism after exposure to the compounds. The overall metabolic responses of yeast to the drug nitisinone and its degradation products were also observed when the results were combined, as shown in Fig. 5a. The mass spectra were processed for baseline correction, peak extraction and normalization, to obtain an aligned matrix for each mass spectrum. PCA was applied on the matrix to distinguish different of treatment of NTBC or its metabolites and to figure out the significant variables among the different samples. After executing PCA to the obtained MS data, separation of clusters in PCA plots for differently treated samples can be clearly seen, indicating that highly significant differences exist among the analyzed samples for both positive and negative modes mass spectra. A strong separation between the blank sample and the treated samples was observed, which occurred when comparing the metabolic responses after 30 min (Fig. 5a) and 120 min of incubation (Fig. 6a). In all cases, the blank was separated from the exposed samples. As seen in Fig. 5, there was a significant separation for the yeast metabolic responses after exposure to CHD. Taking into account the metabolic responses of yeast to NTFA and ATFA, it can be concluded that there is an overlap between these two compounds (Fig. 5a), most likely because of the similarities in the structures of these compounds. This relationship may also be explained by the assumption that ATFA is formed during the degradation of NTFA, which is consistent with the hypothesis presented in the degradation studies in "Degradation of NTBC and its metabolites".

The yeast metabolic response after NTBC treatment was different from the response induced by NTBC metabolites after 30 min of incubation. Nevertheless, it should be noted that after 120 min, this response overlapped with those generated by the yeast treatment with ATFA and NTFA (Fig. 5a), confirming the assumption that NTBC degradation produces the metabolites ATFA and NTFA. The responses obtained after exposure to yeast CHD were separated from the rest of the graph and did not show a common correlation, which demonstrates the distinct properties of this compound. After the configuration of the graph by varying between variance values, it was possible to distinguish the metabolic response to ATFA from that to NTFA. This was possible for samples obtained both after 30 min (Fig. 5b) and after 120 min of incubation (Fig. 6b). The clusters of metabolic responses to each compound are indicated on the graphs as circles. If the ellipses on the PCA plot do not overlap, these groups form separate clusters. The variability of the matrix was confirmed by comparing the metabolic responses of blank samples obtained after 30 and 120 min of incubation, which were significantly different in the concentrations of endogenous compounds (Sect. 2SM, Fig. 10SM). This was taken into account when processing the results; therefore, each change in analyte concentration was determined with respect to the blank prepared in parallel in each series of samples.

## Conclusions

In metabolic profiling, no significant changes in the metabolic responses of the l-tyrosine catabolism pathway were found in yeast exposed to nitisinone. However, NTBC degradation products induced changes in yeast metabolic profiles. These compounds had a reducing effect on the l-TYR, l-PHE, and l-TRF concentrations in comparison to the blank sample. The strongest effect was exerted by CHD, which reduced the content of l-TYR by as much as 34%, that of l-PHE by 51%, and that of l-TRF by 59% after 120 min of yeast incubation. The CHD was the most stable compound among the NTBC metabolites under study, which explains its strong reducing effect on endogenous compounds in yeast. The results obtained from studies on the degradation of nitisinone and its metabolites were confirmed by metabolic profiling and non-targeted analyses. During the degradation of NTFA, the derivative ATFA is formed, which enhances the effect on changes in the l-TYR metabolic pathway. The synergistic action was confirmed by PCA, which demonstrated the statistically significant similarity of yeast metabolic responses exposed to ATFA and NTFA. The PCA also confirmed the distinct effect of CHD—the results obtained after exposure to yeast CHD were separated from the rest of the graph and did not show a common correlation. Based on the results of NTBC degradation kinetics studies, information was obtained on the metabolic rate of this drug in the model organism and on the formation rates of the metabolites ATFA and NTFA during this process. The registered changes comprise increased and decreased concentrations within the same pathways, suggesting that nitisinone metabolites have complex, wide-ranging effects on metabolism. This study provides important new insights into the influence of these compounds on the concentrations of l-TRF, l-PHE, and l-TYR, which play a key role in neurotransmitter biosynthesis. This work makes a significant original contribution to the understanding of the metabolism of nitisinone and appears to be the first to find that the NTBC degradation produces its derivatives ATFA and NTFA, and yet additionally, NTFA is metabolized to ATFA. The results of this study will greatly contribute to further investigations on the effects of these metabolites on the human body and the mechanism of action of nitisinone, which is especially important in the planning of effective therapies for diseases associated with metabolic disorders.

## Supplementary Information

Supplementary Information.

## Data Availability

The raw datasets are available in the MassIve database at ftp://massive.ucsd.edu/MSV000091104/. The datasets used and/or analyzed during the current study are available from the corresponding author upon reasonable request.
