# Supplementary material for: Metabolic profiles and fingerprints for the investigation of the influence of nitisinone on the metabolism of the yeast Saccharomyces cerevisiae
Source: Sci Rep. 2023 Jan 26;13:1473. doi: 10.1038/s41598-023-28335-3 (PMC9879944; doi:10.1038/s41598-023-28335-3)
Supplement: Supplementary file 1 — Supplementary Information. [file 41598_2023_28335_MOESM1_ESM.pdf]

**Supplementary Materials**

# Metabolic profiles and fingerprints for the investigation of the influence of nitisinone on the metabolism of the yeast Saccharomyces cerevisiae

Hanna Barchanska [a], Joanna Płonka [a], Paulina Nowak [a], Marianna Kostina-Bednarz [a,b] *

[a] *Department of Inorganic Chemistry, Analytical Chemistry and Electrochemistry, Faculty of Chemistry, Silesian University of Technology, B. Krzywoustego 6, 44-100 Gliwice, Poland*

[b] *Biotechnology Centre, Silesian University of Technology, B. Krzywoustego 6, 44-100 Gliwice, Poland*

* Corresponding author.
*E-mail address*: marianna.kostina-bednarz@polsl.pl

Table 1SM. Physico-chemical properties of NTBC and its main metabolites.

| Systematic name | Structure of a compound | logP | p$K_a$ |
|---|---|---|---|
| **NTBC**<br>2-(2-nitro-4-trifluoromethylbenzoyl)-1,3-cyclohexanedione | 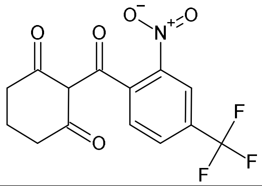 | 3.13 | 7.44 |
| **ATFA**<br>2-amino-4-(trifluoromethyl)benzoic acid | 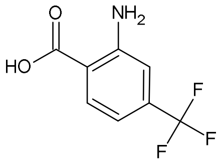 | 2.33 | 4.71 |
| **NTFA**<br>2-nitro-4-(trifluoromethyl)benzoic acid | 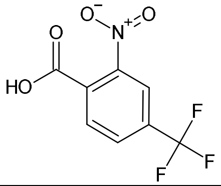 | 2.45 | 1.14 |
| **CHD**<br>cyclohexane-1,3-dione | 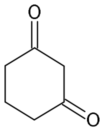 | 0.82 | 8.18 |

## 1SM. Optimization of the extraction process

According to the literature, there is still no consensus on which extractor is the most appropriate one for the extraction of catecholamine from a yeast matrix prior to LC analysis. Based on the pre-experiment, 20 mM FA in methanol, 39% AA in water, 20 mM AA in acetonitrile, and 20 mM FA in acetonitrile were selected as extraction solvents, and their extraction efficiencies were evaluated in the presented study. As seen in Figure 1SM, the solution with 20 mM FA in acetonitrile provided the highest extraction efficiency of all analytes among all tested solvents. Therefore, for the following experiments, 8 mM FA in acetonitrile was used, which corresponds to the mobile phase component used for chromatographic analysis.

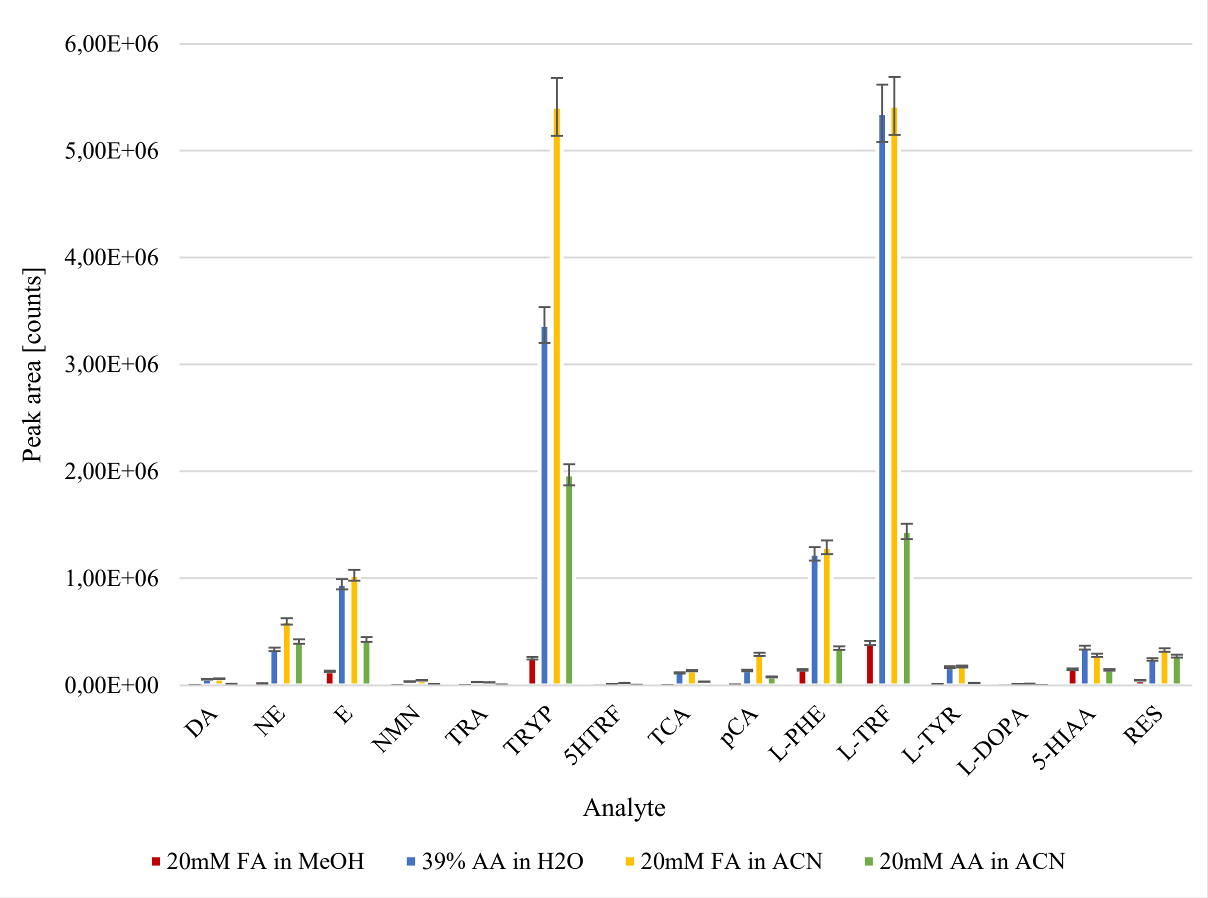


Fig. 1SM. Selection of the extraction solvent. Data are shown as a mean ± CV, n = 3.

Table 2SM. Time-scheduled gradient elution program.

| Type of analysis | Time [min] | Mobile phase composition | | Flow rate [mL/min] | Temperature [°C] | Injection volume [µL] |
|---|---|---|---|---|---|---|
| | | 8 mM FA in ACN [%] | 8 mM FA in $H_2O$ [%] | | | |
| **Determination of NTBC and its metabolites** | 0.0 | 50 | 50 | 0.8 | 40 | 10 |
| | 5.0 | 90 | 10 | | | |
| | 7.0 | 50 | 50 | | | |
| **Determination of L-TYR, L-PHE, L-TRF and its metabolites** | 0.0 | 2 | 98 | 0.8 | 20 | 10 |
| | 4.5 | 2 | 98 | | | |
| | 15.0 | 100 | 0 | | | |
| | 15.1 | 2 | 98 | | | |
| | 22.0 | 2 | 98 | | | |
| **Non-targeted analysis** | 0.0 | 10 | 90 | 0.5 | 25 | 20 |
| | 15.0 | 100 | 0 | | | |
| | 20.0 | 10 | 90 | | | |

Table 3SM. Method validation data for NTBC and its metabolites.

| Analyte | Range [µg/mL] | [$R^2$] | LOD[a] [µg/mL] | LOQ[b] [µg/mL] | ME[c] [%] | Concentration [µg/mL] | CV[d] [%] | Recovery ± SD[e] [%] |
|---|---|---|---|---|---|---|---|---|
| NTBC | 0.2 – 8.0 | 0.9850 | 0.0069 | 0.0210 | 74.9 | 1.0 | 3.2 | 94.1 (5.6) |
| | | | | | | 4.0 | 4.3 | 112.7 (6.3) |
| | | | | | | 8.0 | 2.3 | 85.2 (4.5) |
| ATFA | 0.2 – 8.0 | 0.9941 | 0.0014 | 0.0042 | 134.6 | 1.0 | 4.6 | 64.5 (3.4) |
| | | | | | | 4.0 | 2.8 | 42.9 (2.8) |
| | | | | | | 8.0 | 6.1 | 67.0 (4.2) |
| NTFA | 0.2 – 8.0 | 0.9931 | 0.0025 | 0.0077 | 62.1 | 1.0 | 4.0 | 51.9 (2.2) |
| | | | | | | 4.0 | 3.5 | 72.6 (2.7) |
| | | | | | | 8.0 | 4.0 | 36.4 (1.5) |
| CHD | 0.2 – 8.0 | 0.9937 | 0.0587 | 0.1779 | 8.3 | 1.0 | 3.5 | 82.8 (2.9) |
| | | | | | | 4.0 | 3.2 | 35.1 (3.5) |
| | | | | | | 8.0 | 2.3 | 69.8 (4.1) |

a) LOD – limit of detection. b) LOQ – limit of quantification. c) ME – matrix effect.
d) CV – coefficient of variation. e) SD – standard deviation.

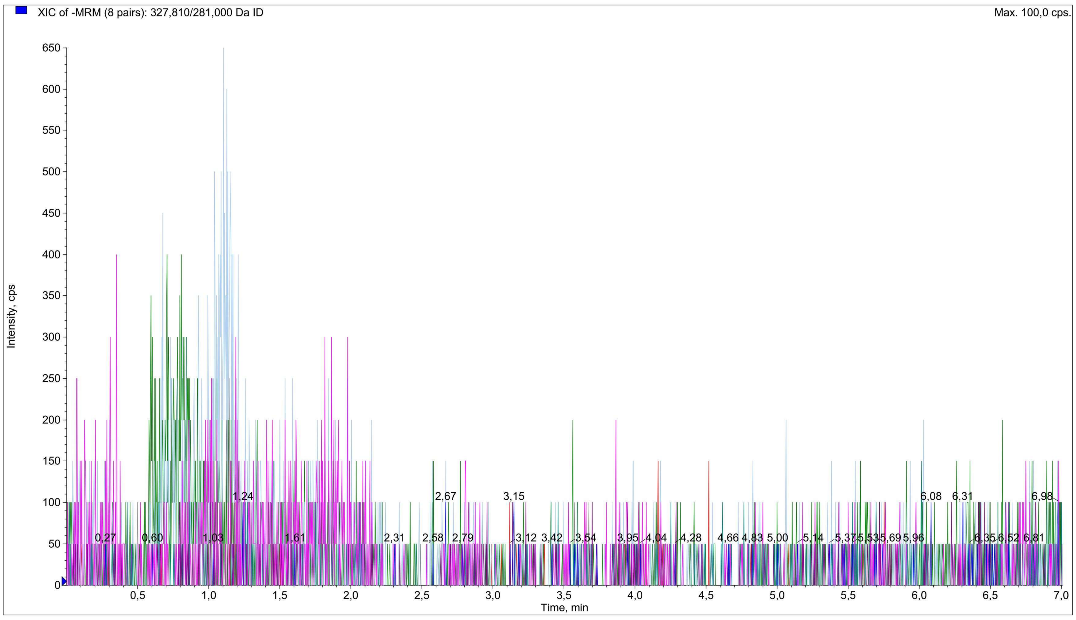


Fig. 2SM. Chromatogram of blank sample extract analyzed by the method for determination of NTBC and its metabolites.

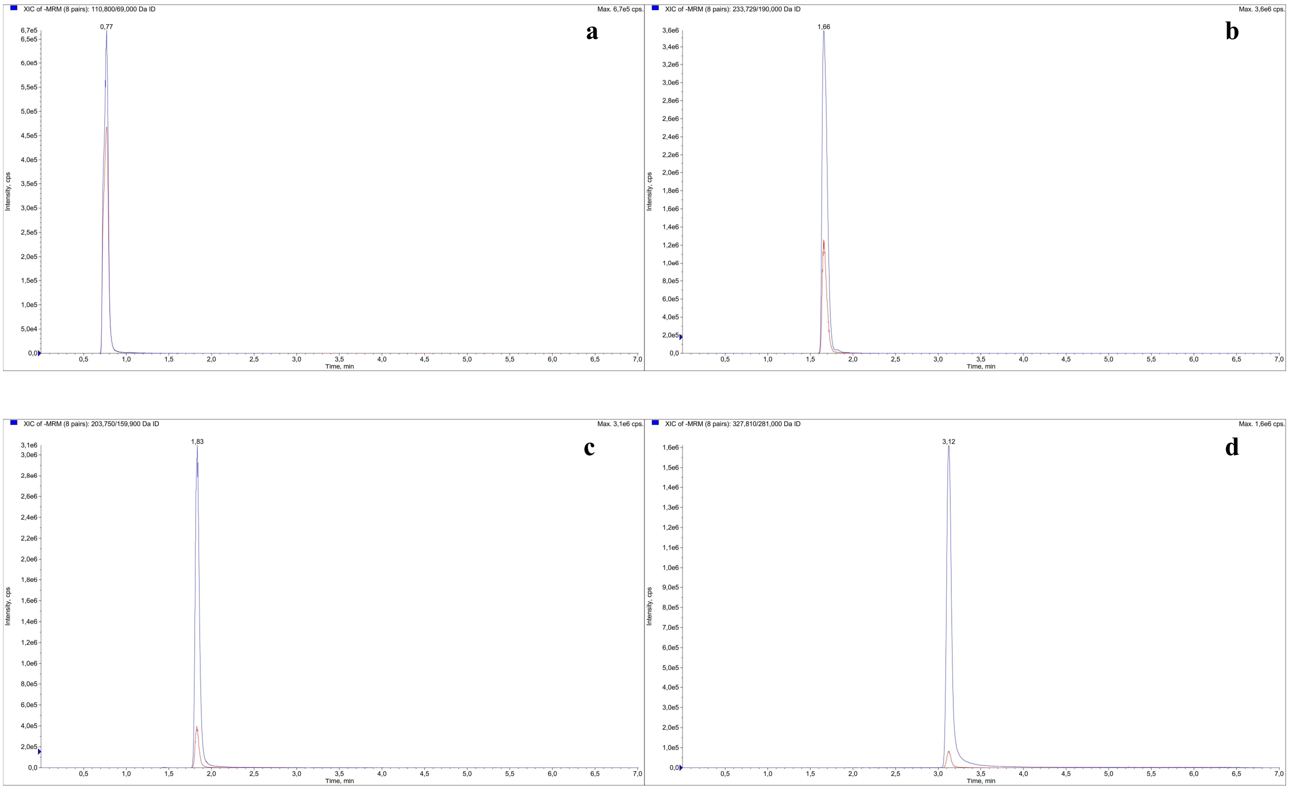


Fig. 3SM. Chromatograms of a matrix solution spiked with standard solutions
**a**) CHD, **b**) NTFA, **c**) ATFA, **d**) NTBC; 10 µg/mL.

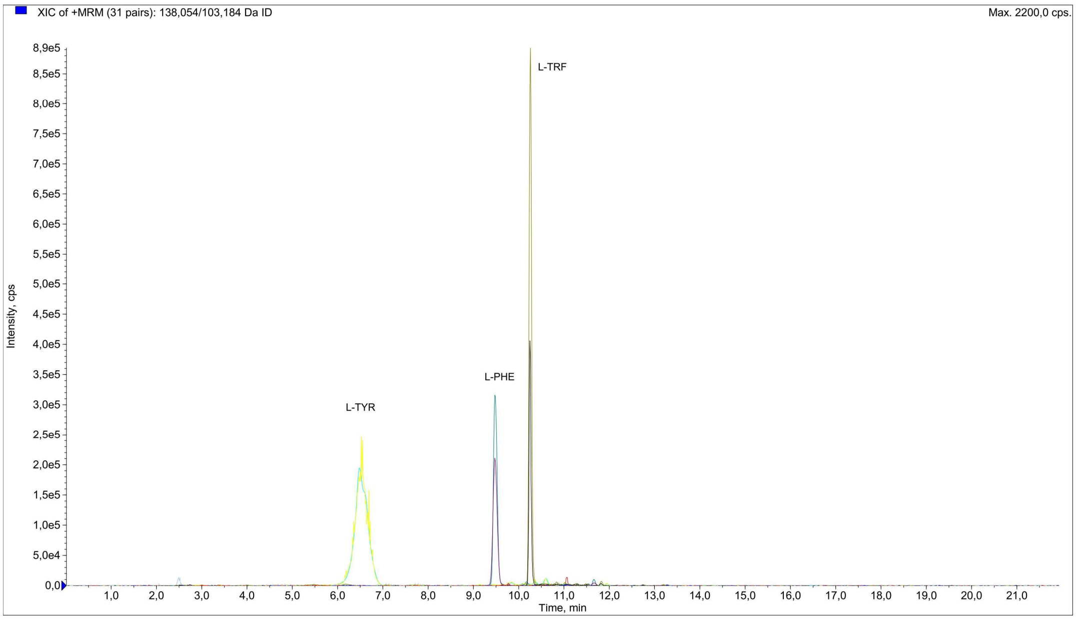


Fig. 4SM. Chromatogram of blank sample extract analyzed by the method for determination of L-TYR, L-PHE, L-TRF and its metabolites.

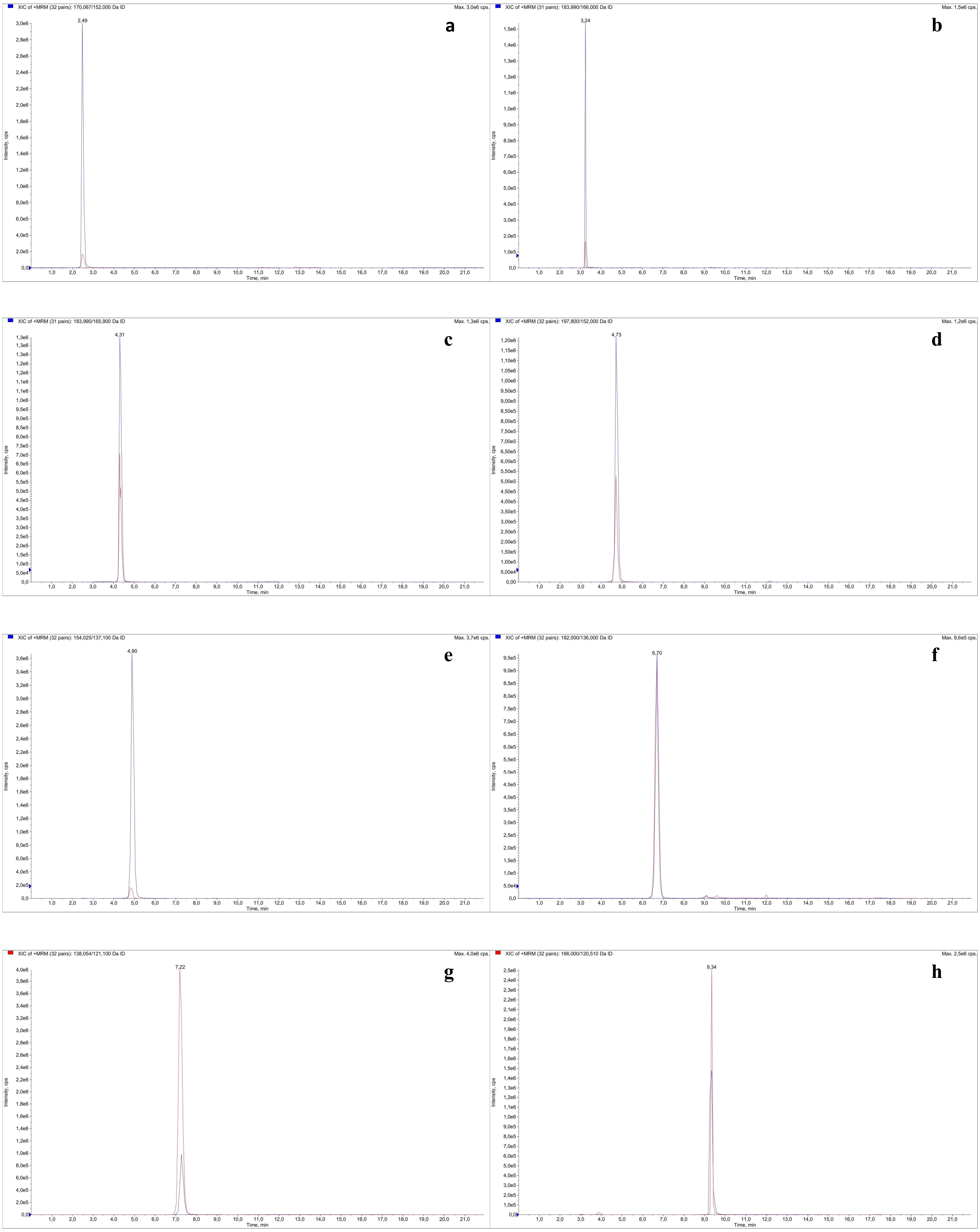


Fig. 5SM. Chromatograms of a matrix solution spiked with standard solutions standards **a**) norepinephrine, **b**) epinephrine, **c**) normetanephrine, **d**) 3,4-dihydroxy-L-phenylalanine, **e**) dopamine, **f**) L-tyrosine, **g**) tyramine, **h**) L-phenylalanine; 10 µg/mL.

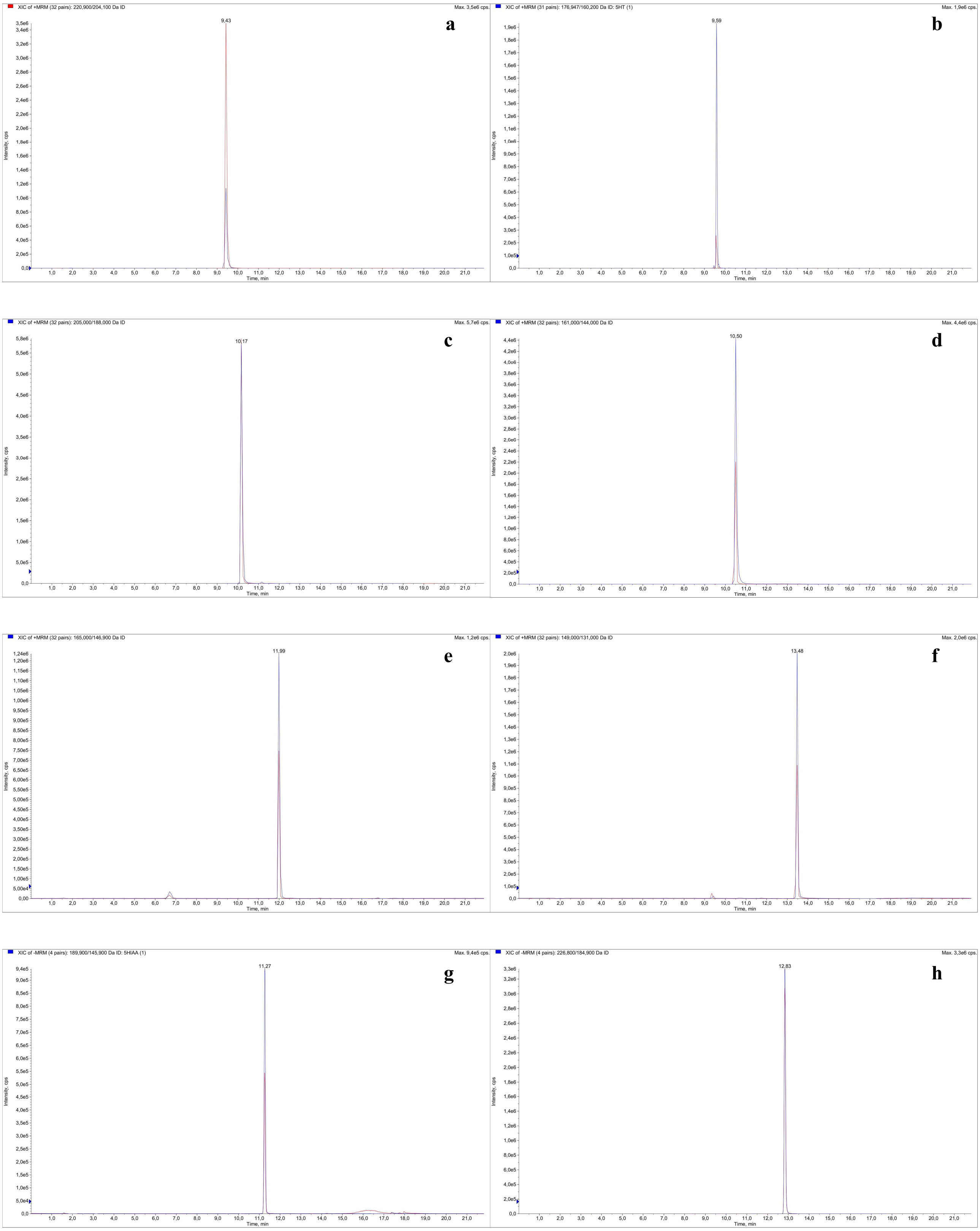


Fig. 6SM. Chromatograms of a matrix solution spiked with standards solutions
**a**) 5-hydroxy-L-tryptophan, **b**) 5-hydroxytryptamine, **c**) L-tryptophan, **d**) tryptamine,
**e**) p-coumaric acid, **f**) trans-cinnamic acid, **g**) 5-hydroxyindole-3-acetic acid, **h**) resveratrol; 10 µg/mL.

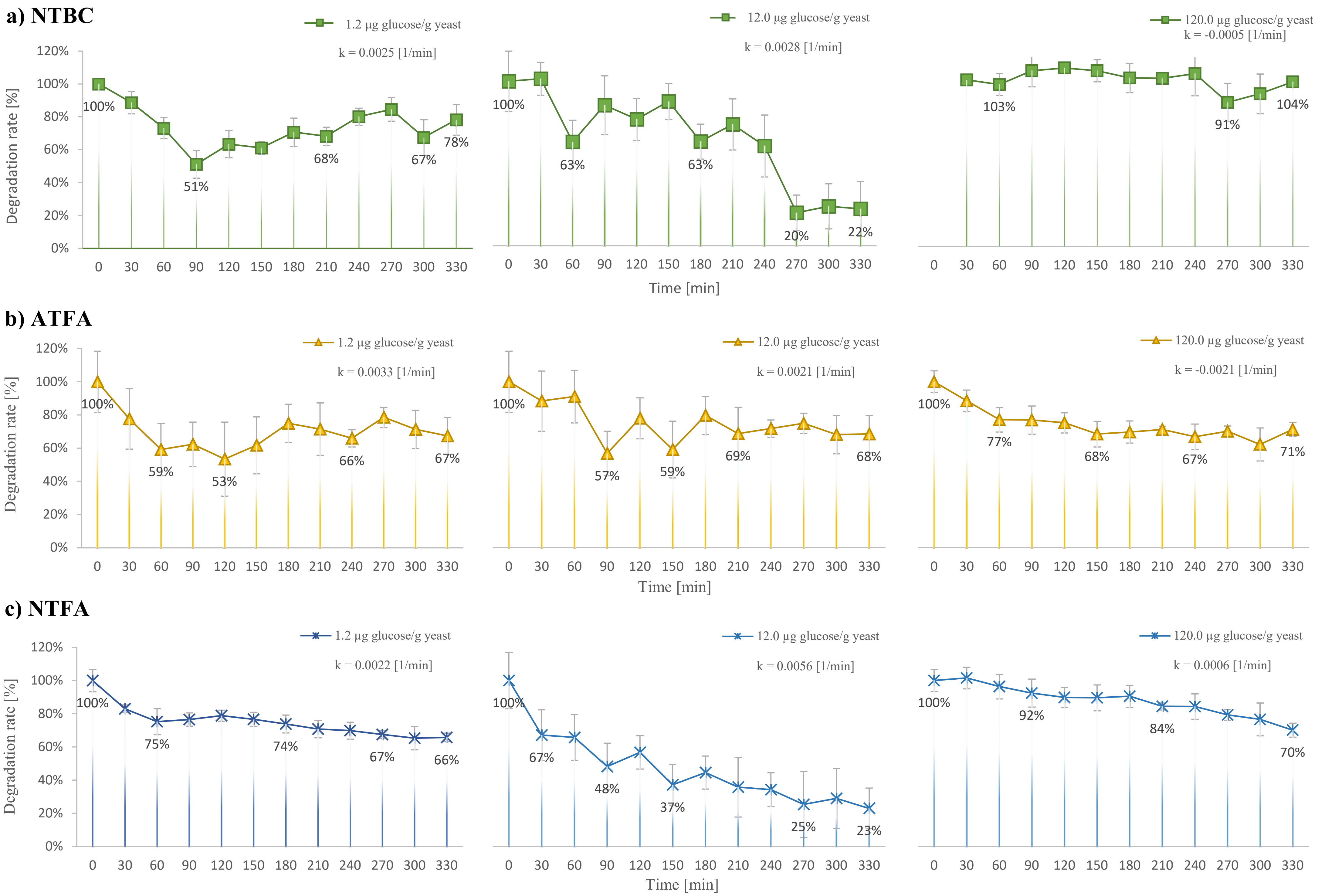


Fig. 7SM.The degradation rate of **a**) NTFA, **b**) ATFA, and **c**) NTFA depends on the amount of glucose added during yeast incubation. Data are expressed as mean (n = 3); error bars represent CV.

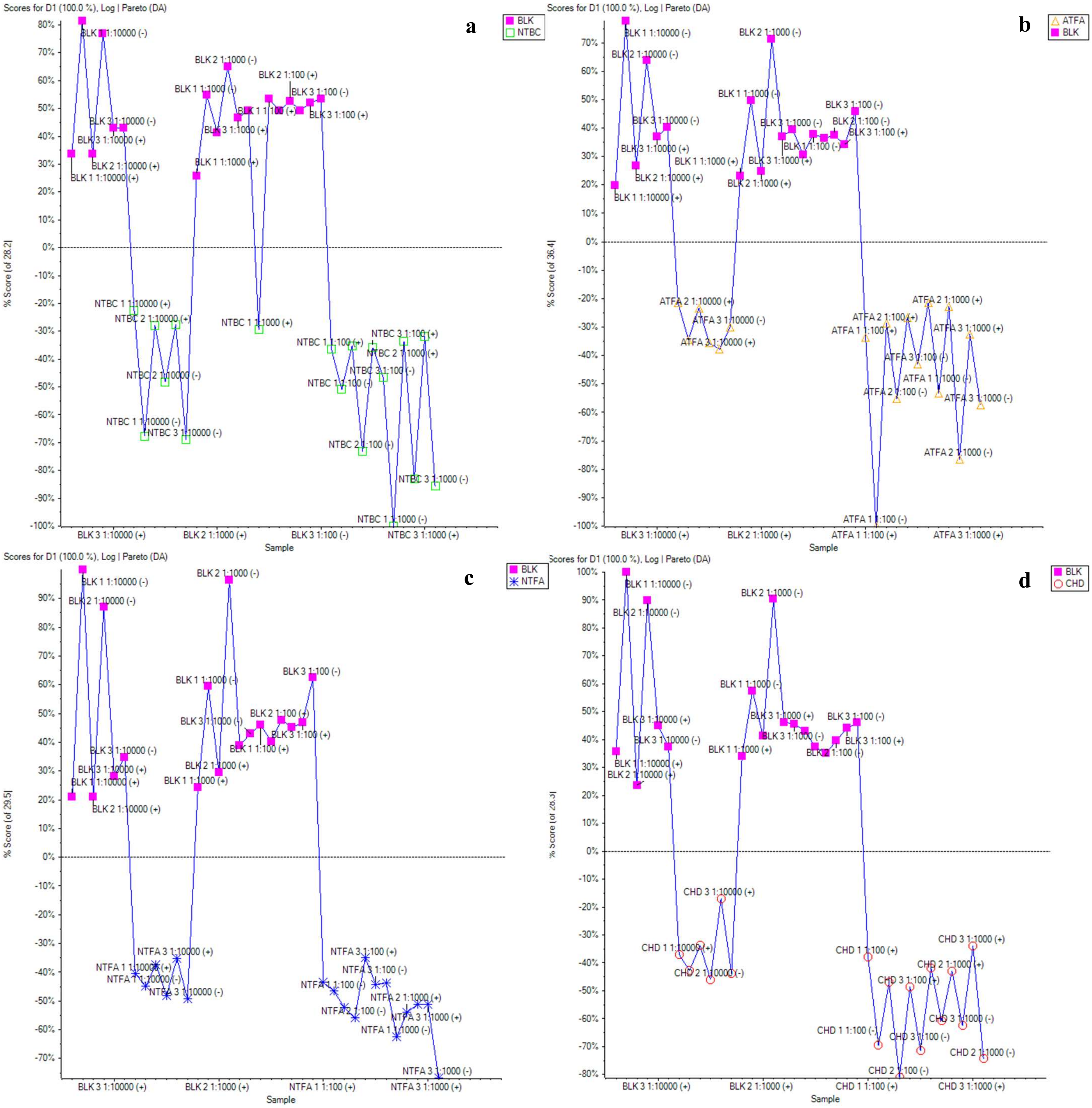


Fig. 8SM. PCA score plots of a data set obtained after 30 minutes of yeast incubation.
a) Comparison of data for NTBC and blank. b) Comparison of data for ATFA and blank.
c) Comparison of data for NTFA and blank. d) Comparison of data for CHD and blank.

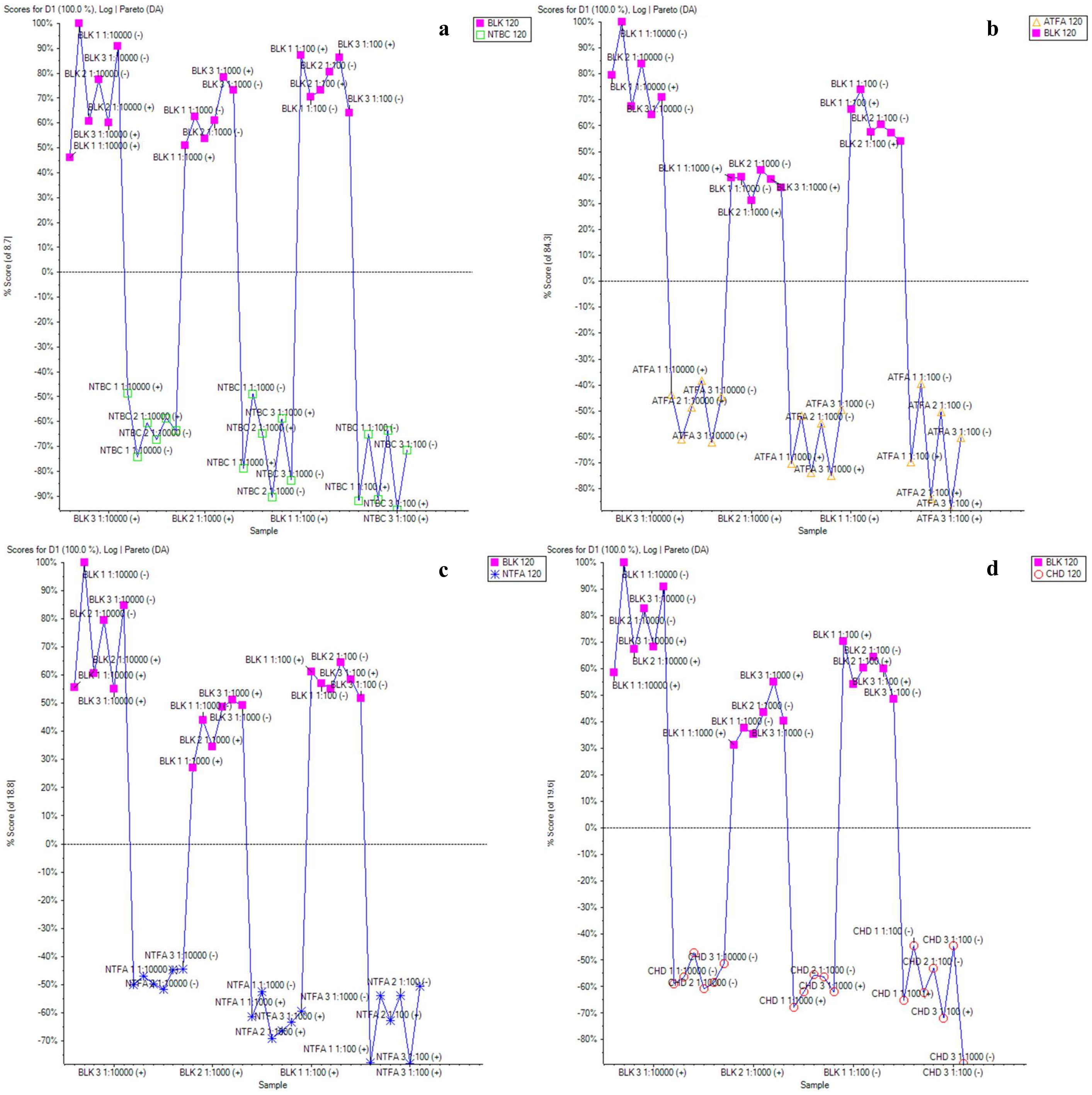


Fig. 9SM. PCA score plots of a data set obtained after 120 minutes of yeast incubation.
a) Comparison of data for NTBC and blank. b) Comparison of data for ATFA and blank.
c) Comparison of data for NTFA and blank. d) Comparison of data for CHD and blank.

**2SM. Visualization of the comparison of blank samples obtained after 30 and 120 minutes of yeast incubation using a PCA score plot.**

A comparison was made between the metabolic responses of the blank samples obtained after 30 and 120 minutes of yeast incubation, Fig. 4SM. The graph shows a significant separation of the results for 30 and 120 minutes, regardless of extract dilution and ionization mode. The noticeable change in the matrix extract during the experiment indicates that the model organism exhibited various metabolic processes during incubation. Because of this, the results obtained from the experiments are difficult to interpret, and the validation results are not fully satisfactory.

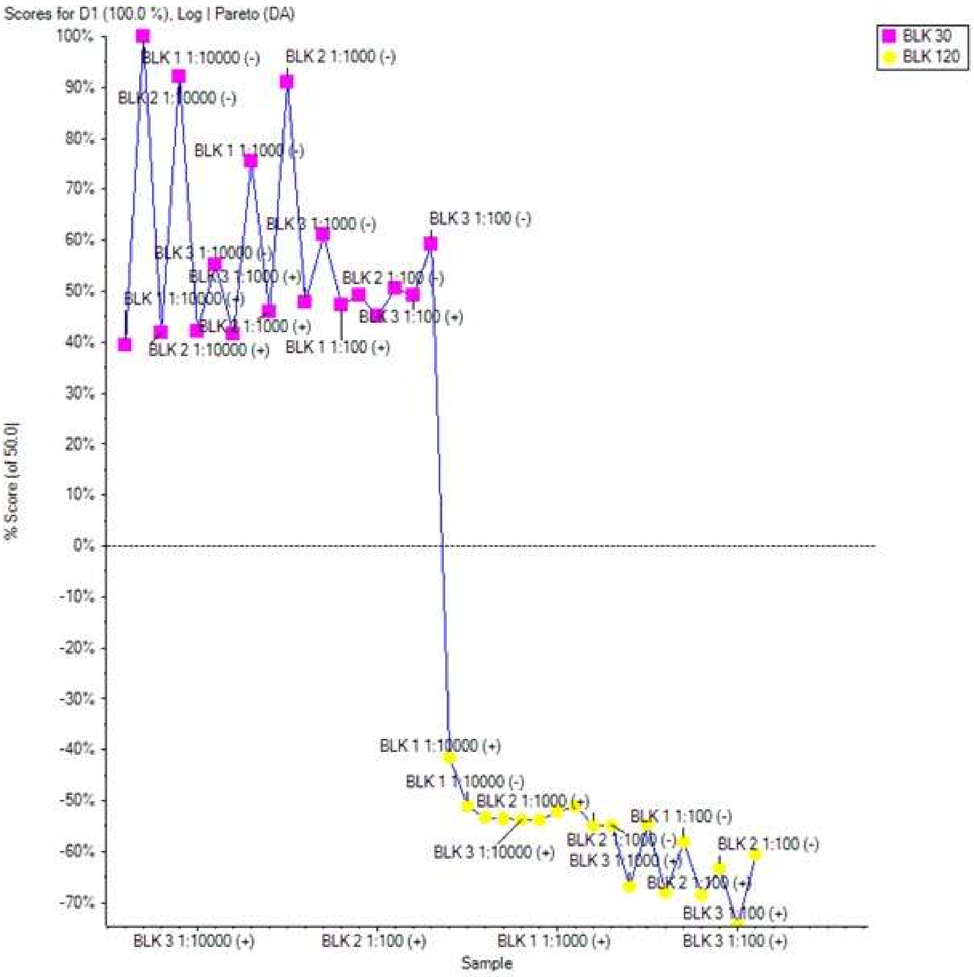


Fig. 10SM. PCA score plot of the blank sample data obtained after 30 and 120 minutes of yeast incubation.
